# Recruiting ahead of target: What worked in the REEACT trial?

**DOI:** 10.1186/1745-6215-12-S1-A126

**Published:** 2011-12-13

**Authors:** Puvan Tharmanathan, Gwen Brierley, Elizabeth Littlewood, Phil Andersen, Simon Gilbody

**Affiliations:** 1 Department of Health Sciences, University of York, York, YO10 5DD, UK; 2 School of Social & Community Medicine, Bristol University, Bristol, BS8 2PS, UK

## Background

Recruitment to randomised controlled trials (RCTs) is a known problem, with many failing to reach recruitment targets [1]. RCTs involving participants with mental health problems often struggle to recruit. This is a particular problem in primary care [2]. The multi-centre REEACT (Randomised Evaluation of the Effectiveness and Acceptability of Computerised Therapy) trial led by the University of York [ISRCTN91947481; http://www.reeact.org.uk] recently completed recruitment ahead of target. It recruited primary care patients with depression using two strategies - database screening (DS) to identify potential eligible recruits to target, and traditional GP direct referral (DR) from face-to-face consultations. These strategies were used in combination in the hope of expediting recruitment, which was achieved. With recruitment complete, we examined this recruitment strategy in more detail.

## Materials and methods

We tabulated the overall contributions of each method of recruitment to the overall number of participants and examined the trend in recruitment over the course of the trial. We checked for statistically significant differences in the baseline characteristics of participants recruited via each method. In order to see if there was regional variation in use of recruitment methods, we compared the number of participants recruited from each method by study site. The conversion rate to trial participants for those patients identified through each method was also compared.

## Results

The majority of participants (72%) were recruited via DR. The participants recruited through DS were older on average, and had a higher probability of having had a previous episode of depression. The proportion of participants entering the trial via each method was consistent with the overall recruitment figures across all sites except York, where the contribution from DS was slightly higher. The proportion of participants entering the trial through each referral method remained consistent from about a year before the end of recruitment. A higher proportion of DRs assessed for inclusion converted into participants and a lower proportion were ineligible as compared to those identified via DS.

## Conclusions

The pragmatic design of the REEACT trial resulted in target recruitment ahead of schedule. A detailed examination of the recruitment trend suggests that DR was a more effective method of recruitment, although the use of DS has been a favoured tool in primary care trials. The findings from the REEACT suggest that DRs may be a better strategy when recruiting patients with depression in the primary care setting.
